# Critical role of insulin-like growth factor binding protein-5 in methamphetamine-induced apoptosis in cardiomyocytes

**DOI:** 10.3892/mmr.2014.2572

**Published:** 2014-09-16

**Authors:** KA-PUI LEUNG, YI-HONG QU, DONG-FANG QIAO, WEI-BING XIE, DONG-RI LI, JING-TAO XU, HUI-JUN WANG, XIA YUE

**Affiliations:** 1 Department of Forensic Medicine, Southern Medical University, Guangzhou, Guangdong 510515, P.R. China; 2 Department of Legal Medicine, Osaka City University Medical School, Abeno, Osaka 545-8585, Japan

**Keywords:** methamphetamine, insulin-like growth factor binding protein-5, small interfering RNA, cardiotoxicity, apoptosis

## Abstract

Methamphetamine (MA) is a highly abused amphetamine-like psychostimulant. At present, the mechanisms underlying MA-induced cardiotoxicity are poorly understood. The cardiotoxic effects have yet not been clearly elucidated with respect to the apoptotic pathway. Insulin-like growth factor binding protein-5 (IGFBP5) is important for cell growth control and the induction of apoptosis. The aim of the present study was to analyze whether IGFBP5 is involved in MA-induced apoptosis as a novel target. MA-induced apoptosis was observed in neonatal rat ventricular myocytes (NRVMs) in a concentration-dependent manner using a terminal deoxyribonucleotide transferase-mediated dUTP nick end-labeling assay. Using reverse transcription polymerase chain reaction and western blotting, MA was demonstrated to induce concentration-dependent increases in the expression of IGFBP5. Silencing IGFBP5 with small interfering RNA significantly reduced apoptosis and suppressed the expression of caspase-3 in NRVMs following treatment with MA. To the best of our knowledge, the present study provided the first evidence suggesting that IGFBP5 is a potential therapeutic target in MA-induced apoptosis *in vitro*, providing a foundation for future *in vivo* studies.

## Introduction

Methamphetamine (MA) is a highly addictive psychostimulant that belongs to the phenethylamine and amphetamine class of psychoactive drugs. Its hydrochloride is a colorless transparent crystal, commonly termed ‘ice’. MA is a potent and addictive drug of abuse that has long been associated with a myriad of adverse effects. It is known to cause harm to the human brain, heart, liver and kidney. The majority of studies have focused on the neurotoxicity and neuropsychological deficits caused by MA (1–3). MA was reported to cause widespread apoptosis in the mouse brain, including in the striatum, cortex and hippocampus indicating that apoptosis is an important molecular mechanism involved in MA-induced neurotoxicity (4). Previous studies have demonstrated that MA is able to cause neuropathological alterations in the rodent brain via apoptotic mechanisms similar to those reported in various models of neuronal death (5–7). The accumulated data support that MA-induced cell death is accompanied by activation of several pathways whose roles have been well documented in other models of neuronal apoptosis.

There has been increasing concern that prescription stimulant use may be linked to adverse cardiovascular complications. Chronic use or acute overdose of MA may lead to, not only psychological but also physical harm, primarily consisting of cardiovascular damage (8). Previous studies have demonstrated evidence of MA-associated acute and chronic cardiovascular pathology (9). To date, MA-induced acute myocardial infarction has been overwhelmingly reported (10–15). In addition to this, much of the evidence for MA-induced cardiomyopathy is also present in humans. Cardiomyopathy is typically a chronic disease of gradual onset that is often associated with chronic MA use (16–18). Cardiomyocyte apoptosis is important in the progression of heart failure. In patients with end-stage cardiomyopathy, loss of cardiomyocytes due to apoptosis is observed leading to the progression of cardiac dysfunction and ultimately heart failure (19–22).

However, whether MA is involved in regulating cardiomyocyte apoptosis in MA-induced myocardial injury remains to be elucidated. The mechanisms underlying MA-induced cardiotoxicity have not yet been clearly elucidated with respect to the apoptotic pathway. In addition, no effective treatment exists for the adverse effects of MA. Therefore, in the present study, it was hypothesized that identifying a novel target to inhibit apoptosis could partially reduce the symptoms caused by MA and protect cardiomyocytes from apoptosis.

To elucidate the possible mechanism underlying MA-induced toxicity, our laboratory performed gene expression microarray analysis and revealed that insulin-like growth factor binding protein-5 (IGFBP5) was significantly upregulated in cells (data not shown). Insulin-like growth factor binding proteins comprise a family of proteins that bind and regulate the functions of insulin-like growth factors (IGFs). IGFBP5 is involved in apoptotic processes in various cell types. In prostate and mammary glands and in the rat brain following hypoxic ischemic injury, increased expression of IGFBP5 has been associated with apoptotic processes (23–25).

The aim of the present study was to investigate cardiomyocyte apoptosis induced by MA. It was hypothesized that IGFBP5 may also be involved in the regulation of cardiomyocyte apoptosis induced by MA. However, it is not known whether silencing IGFBP5 is able to protect against the loss of cardiomyocytes due to apoptosis. Silencing through small interfering (si)RNA is a natural mechanism for the suppression of specific gene activity that is highly conserved in evolution. The use of siRNAs in mammals has been extremely useful for understanding the biological role of several genes using a relatively simple and efficient technique (26). In the present study, siRNA-mediated gene silencing was performed to investigate the knockdown of IGFBP5 expression.

## Materials and methods

### Cell culture

NRVMs were prepared from 0–1 day-old neonatal Sprague-Dawley rats (Experimental Animal Center of Southern Medical University, Gangzhou, Guangdong, China). Rats were sacrificed by immersion in 75% alcohol. Ventricles were removed and washed in Hank’s solution, then minced and incubated with 0.25% trypsinase at 4°C for 12–16 h. Dulbecco’s modified Eagle’s medium (Sigma-Aldrich, Shanghai, China) containing 10% fetal bovine serum (Corning Inc., New York, NY, USA) was added to terminate digestion for 5 min at 37°C. The supernatant was discarded. Hank’s solution (25 ml; Gibo-BRL, Paisley, UK) was supplemented with 25 mg collagenase type II (Sigma-Aldrich) and 125 mg bovine serum albumin (Roche Applied Science, Indeanapolis, IN, USA) as digestive solution. Digestive solution was added and placed in a water bath for 1 min at 37°C. The supernatant was discarded. Subsequently, fresh digestive solution was added and placed in a water bath on the top of a hot plate stirrer stirring the tissue fragments with a magnetic bar for 15 min at 37°C and the supernatant was collected. The latter digestion step was repeated four times. Cells in the supernatant were isolated by centrifugation for 10 min at 2,000 × g at room temperature. In order to reduce fibroblast contamination, cells resuspended in NRVMs culture medium were pre-plated for 1 h. The supernatant was aspirated gently and cells were plated onto six-well plates. Cells were incubated at 37°C in a humidified atmosphere containing 5% CO_2_ during the experiments. All animal procedures were conducted in accordance with the National Institutes of Health Guide for the Care and Use of Laboratory Animals (Bethesda, MA, USA) and were approved by the Animal Care and Use Committees of the Southern Medical University.

### Transfection of IGFBP5 siRNA

NRVMs were transfected with siRNA using Lipofectamine 2000 reagent (Invitrogen, Shanghai, China), according to the manufacturer’s instructions. NRVMs were grown to 80–90% confluency. The cells were then transfected with IGFBP5 siRNA and Lipofectamine for 6 h in OptiMEM (Gibco-BRL, Paisley, UK). The siNC was added as a nonspecific control. The complex medium was replaced after 6 h incubation at 37°C with the same volume of fresh culture medium. The cells were maintained in an incubator for 48 h. At 48 h, NRVMs of the MA-exposure groups were treated with 1.5 mM MA for 48 h.

The following two sequences targeting IGFBP5 by the siRNA method were used: siIGFBP5-1, 5′-CGC GTC CCC GGA AGG AAT TCT GGA AGA TAT TTC AAG AGA ATA TCT TCC AGA ATT CCT TCC TTT TTG GAA AT-3′; siIGFBP5-2, 5′-CGC GTC CCC GGA AGA TAT GCC TGT GGA TCC TTC AAG AGA GGA TCC ACA GGC ATA TCT TCC TTT TTG GAA AT-3′.

### RNA extraction and quantitative (q)PCR

Total RNA was extracted using RNAiso Plus (Takara Bio, Inc., Shiga, Japan) according to the manufacturer’s instructions. The concentration and OD260/OD280 value of total RNA were measured using a Beckman Coulter DU 520UV/Vis spectrophotometer (Beckman Coulter, Miami, FL, USA). Reverse transcription was conducted starting from 1 μg of total RNA using a PrimeScript RT reagent kit with gDNA Eraser (Takara Bio, Inc.) and excluded any potential genomic DNA contamination. The mRNA levels were measured by RT-PCR with an Applied Biosystems 7500 fast real-time PCR system (Applied Biosystems, Foster City, CA, USA) using SYBR Premix Ex Taq II (Takara Bio, Inc.). The PCR cycling conditions were as follows: 2 min at 50°C, 30 sec at 95°C, 40 cycles of 15 sec at 95°C and 34 sec at 60°C, followed by a dissociation stage. The IGFBP5 fragment was amplified using the following primers: IGFBP5, forward 5′-ATG AAG CTG CCG GGC-3′ and reverse 5′-TCA ACG TTA CTG CTG TCG AAG-3′. RNA content was normalized to 18S ribosomal RNA and relative changes in gene expression were quantified using the threshold cycle (2^−ΔΔCt^) method with RQ software (Applied Biosystems).

### Western blotting

Total protein was extracted for western blotting. Samples were homogenized in ice-cold RIPA buffer (Santa Cruz Biotechnology, Inc., Santa Cruz, CA, USA) containing protease inhibitors. The protein concentration of the samples was determined using a BCA protein assay kit (Thermo Fisher Scientific, Waltham, MA, USA). Protein samples were separated by sodium dodecyl sulfate polyacrylamide gel electrophoresis and transferred onto polyvinylidene difluoride membranes (Millipore, Billerica, MA, USA). Non-specific binding was blocked with 5% non-fat skim milk in Tris-buffered saline with Tween 20 (TBST) at room temperature for 1 h. Membranes were incubated with rabbit polyclonal anti-IGFBP5 antibody (1:1,000; Abcam, Cambridge, UK) and rabbit polyclonal anti-caspase-3 antibody (1:200, Santa Cruz Biotechnology, Inc.) at 4°C overnight. Following being washed with TBST, membranes were reacted with horseradish peroxidase-conjugated goat anti-rabbit IgG (1:2,000; Amersham Biosciences, Tokyo, Japan) at room temperature for 1 h. The bound secondary antibodies were visualized with Thermo Scientific Pierce ECL Western Blotting Substrate (Thermo Scientific Pierce, Rockford, IL, USA) according to the manufacturer’s instructions. Signal intensities of bands were analyzed and the relative protein levels were calculated by comparison with the quantity of β-actin as a loading control.

### Evaluation of apoptosis

Apoptotic cells were quantified using a terminal deoxyribonucleotide transferase-mediated dUTP nick end-labeling (TUNEL) assay (Roche Applied Science, Indianapolis, IN, USA) according to the manufacturer’s instructions. NRVMs were fixed in 4% paraformaldehyde (Dingguo Changsheng, Beijing, China) in fresh phosphate-buffered saline (pH 7.4; Dingguo Changsheng) at 4°C for 1 h, incubated with fluorescein-conjugated terminal deoxynucleotidyl transferase enzyme for 1 h at 37°C in the dark, and then mounted with 4′,6′-diamidino-2-phenylindole (Dingguo Changsheng) for nuclear counterstaining. Cross-sections were imaged (20× and 40× objective) using a fluorescent microscope (Nikon, Tokyo, Japan). The apoptotic rate was calculated as follows: (number of apoptosis cells / total number of cells) × 100%.

### Statistical analysis

Statistical analyses of the data were performed using SPSS 16.0 software (SPSS, Inc., Chicago, IL, USA). Statistics were performed using one way analysis of variance and Student’s t-test. The results are presented as the mean ± standard deviation and P<0.05 was considered to indicate a statistically significant difference.

## Results

### MA induces apoptosis in NRVMs

**Figure 1:**
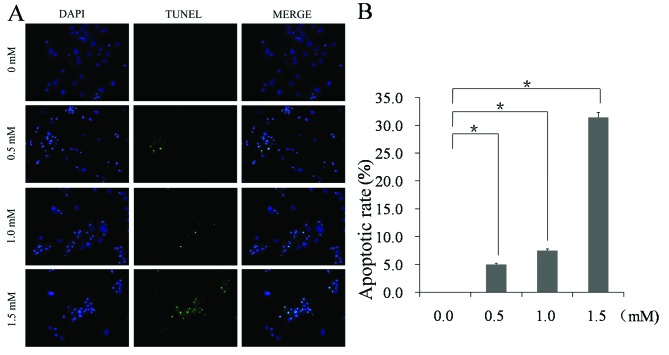
MA induces apoptosis in NRVMs. (A) Effects of MA treatment in NRVMs with varying concentrations (0, 0.5, 1.0 or 1.5 mM) assessed by TUNEL assay. Nuclei were counterstained with DAPI (blue). Apoptotic cells were stained with TUNEL (green). (B) Quantification of the percentage of apoptotic cells using a standard cell counting method with the TUNEL assay. The apoptotic rate was calculated as follows: (number of apoptotic cells / total number of cells) × 100%. The data are presented as the mean ± standard deviation from three independent experiments (n*=*5; ^*^P<0.01). MA, methamphetamine; TUNEL, terminal deoxyribonucleotide transferase-mediated dUTP nick end-labeling; NRVMs, neonatal rat ventricular myocytes; DAPI, 4′,6′-diamidino-2-phenylindole.

A TUNEL assay was performed to detect DNA damage in NRVMs. The results indicated that the apoptotic rate increased in NRVMs exposed to 1.5 mM MA relative to the control cells (31.43±0.50 vs. 0.00±0.00%; n*=*5; P<0.01). No TUNEL-positive cells were observed in the control group, whereas a small amount of TUNEL-positive cells were observed in the 0.5 mM MA group (5.00±0.23%), a moderate amount in the 1.0 mM MA group (7.50±0.30%) and a significantly increased amount of TUNEL-positive cells were observed in the 1.5 mM MA group (Fig. 1). The images show representative TUNEL staining from three independent experiments.

### MA increases IGFBP5 mRNA and protein expression in NRVMs

**Figure 2:**
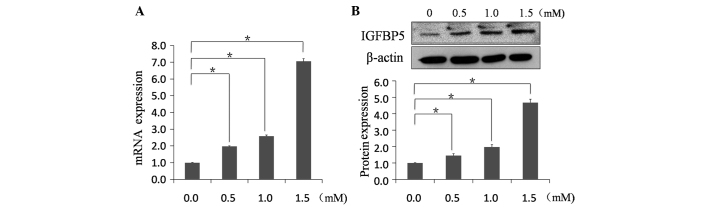
The mRNA and protein expression of IGFBP5 increases in a dose-dependent manner in NRVMs treated with MA for 48 h. NRVMs were incubated with MA at varying concentrations (0, 0.5, 1.0 or 1.5 mM). (A) Total RNA was subjected to reverse transcription polymerase chain reaction. (B) Whole cell lysates were analyzed by western blotting. An antibody against β-actin was used as the loading control. The data are presented as the mean ± standard deviation (n*=*6; ^*^P<0.01). MA, methamphetamine; NRVMs, neonatal rat ventricular myocytes; IGFBP5, insulin-like growth factor binding protein-5.

IGFBP5 mRNA was identified in NRVMs using qPCR (Fig. 2A). Melting curve analysis confirmed the specificity of transcripts of IGFBP5 in NRVMs. To evaluate how MA affects IGFBP5 expression, NRVMs were exposed to varying concentrations of MA. MA increased the mRNA expression of IGFBP5 in a dose-dependent manner. The mRNA expression of IGFBP5 significantly increased in the 1.5 mM MA group by 7.06±0.16 fold (n*=*6; P<0.01). IGFBP5 protein expression was also evaluated in MA-treated NRVMs (Fig. 2B). Immunoblotting using anti-IGFBP5 antibody indicated that the molecular weight of the IGFBP5 protein was 31 kDa. MA increased IGFBP5 protein expression in MA-treated NRVMs to 4.67±0.21 fold (n*=*6; P<0.01).

### siIGFBP5 knockdown of the IGFBP5 protein in NRVMs

**Figure 3:**
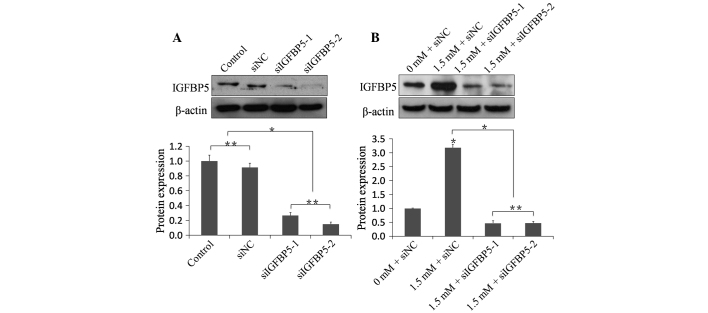
siIGFBP5 silences the protein expression of IGFBP5 in NRVMs. (A) Nonspecific silencing control lane showed no effect of siNC in NRVMs 48 h post-transfection. siIGFBP5-1 and siIGFBP5-2 demonstrated efficient knockdown of IGFBP5 at the protein level. (B) The knockdown efficacy of siIGFBP5 was not affected following treatment with MA. An antibody against β-actin was used as the loading control. The data are presented as the mean ± standard deviation (n*=*6; ^*^P<0.01, ^**^P>0.05). MA, methamphetamine; NRVMs, neonatal rat ventricular myocytes; IGFBP5, insulin-like growth factor binding protein-5; si, small interfering.

The knockdown efficacy of siIGFBP5 on the protein level was investigated. To verify the efficacy of synthetic siRNA-mediated silencing of IGFBP5, two sequences (siIGFBP5-1 and siIGFBP5-2) that target IGFBP5 were selected. The siNC was used as a nonspecific control. Western blot analysis demonstrated that siNC had no silencing effect. The siIGFBP5-1 and siIGFBP5-2 groups exhibited a significant decrease in IGFBP5 compared with the siNC group and the control group, demonstrating that siIGFBP5-1 and siIGFBP5-2 could efficiently knockdown IGFBP5 at the protein level (Fig. 3A). Whether the knockdown efficacy of siIGFBP5 could be affected following MA treatment was also investigated. Following transfection, NRVMs of MA-treated groups were treated with 1.5 mM MA for 48 h. The 1.5 mM + siNC group exhibited a significant increase in IGFBP5 compared with the 0 mM + siNC group (n*=*6; P<0.01). The 1.5 mM + siIGFBP5-1 group and the 1.5 mM + siIGFBP5-2 group exhibited a significant decrease in IGFBP5 compared with the 1.5 mM + siNC group, respectively (n*=*6; P<0.01), demonstrating that siIGFBP5-1 and siIGFBP5-2 could efficiently knockdown IGFBP5 at the protein level even following treatment with MA (Fig. 3B).

### Transfection with siIGFBP5 reduces the apoptotic rate in NRVMs induced by MA

**Figure 4:**
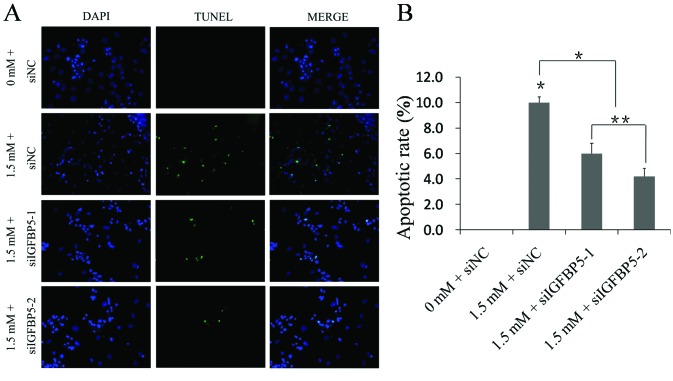
Effect of siIGFBP5 silencing of the IGFBP5 gene on MA-induced apoptosis evaluated by TUNEL staining. (A) Effects of suppressing the IGFBP5 gene in 1.5 mM MA-treated NRVMs assessed by TUNEL assay. The 0 mM + siNC group was pretreated with nonspecific silencing control without MA as the control group. The 1.5 mM + siIGFBP5-1 group and the 1.5 mM + siIGFBP5-2 group were pretreated with two sequences (siIGFBP5-1 and siIGFBP5-2) that target IGFBP5, respectively. Nuclei were counterstained with DAPI (blue). Apoptotic cells were stained with TUNEL (green). (B) Quantification of the percentage of apoptotic cells using a standard cell counting method with the TUNEL assay. The apoptotic rate was calculated as follows: (number of apoptotic cells / total number of cells) × 100%. The data are presented as the mean ± standard deviation from three independent experiments (n*=*5; ^*^P<0.01, ^**^P>0.05). MA, methamphetamine; IGFBP5, insulin-like growth factor binding protein-5; TUNEL, terminal deoxyribonucleotide transferase-mediated dUTP nick end-labeling; DAPI, 4′,6′-diamidino-2-phenylindole; NRVMs, neonatal rat ventricular myocytes; si, small interfering.

Following showing that MA induces apoptosis in NRVMs, it was examined whether silencing the IGFBP5 gene is able to reduce MA-associated impairment *in vitro*. A TUNEL assay was performed to detect DNA damage in NRVMs. The results indicated that the apoptotic rate increased in the 1.5 mM + siNC group relative to the 0 mM + siNC group (10.00±0.45 vs. 0.00±0.00%; n*=*5; P<0.01; Fig. 4). The apoptotic rate decreased in the 1.5 mM + siIGFBP5-1 group and the 1.5 mM + siIGFBP5-2 group relative to the 1.5 mM + siNC group, respectively (6.00±0.80 vs. 10.00±0.45%; 4.20±0.64 vs. 10.00±0.45%; n*=*5; P<0.01; Fig. 4). The images show representative TUNEL staining from three independent experiments.

### Expression of caspase-3

**Figure 5:**
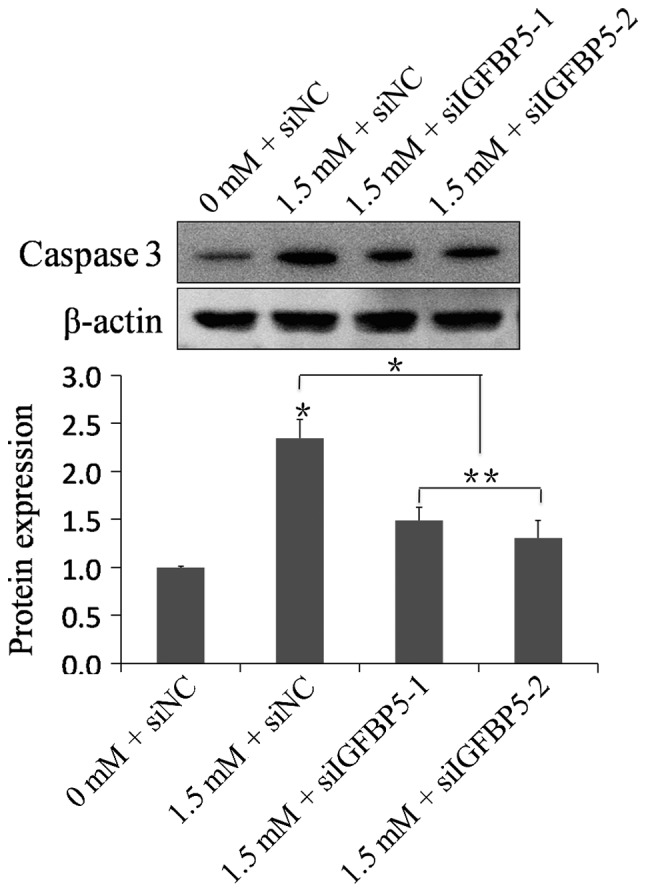
Expression of caspase-3 was determined by western blot analysis. The intensity of each band was quantified by densitometry and the data were normalized using the β-actin signal. The 0 mM + siNC group (control group) was considered the basal level and the other groups were expressed as fold changes compared with the control. The data are presented as the mean ± standard deviation (n*=*6; ^*^P<0.01, ^**^P>0.05). IGFBP5, insulin-like growth factor binding protein-5; si, small interfering.

To investigate whether apoptosis occurred through caspase-3, the expression of caspase-3 was analyzed by western blotting. Compared with the 0 mM + siNC group, the expression of caspase-3 was significantly increased in the 1.5 mM + siNC group (n*=*6, P<0.01). The 1.5 mM + siIGFBP5-1 group and the 1.5 mM + siIGFBP5-2 group exhibited a significant decrease in caspase-3 expression compared with the 1.5 mM + siNC group, respectively (n*=*6, P<0.01; Fig. 5). Silencing IGFBP5 with siRNA significantly suppressed the expression of caspase-3 in NRVMs following MA treatment.

## Discussion

The present study examined whether silencing IGFBP5 is able to protect against the loss of cardiomyocytes due to MA-induced apoptosis. The accumulated evidence supports the idea that MA can lead to the death of cells via apoptosis. MA is known to trigger neuronal, splenic and thymic damage via apoptosis (27,28). The cardiotoxic action of MA has been of particular interest since standardized doses consistently produces myocardial lesions (29,30). To date, studies have focused on MA abusers that almost always have severe damage in their cardiovascular system, yet less is known regarding cardiac damage via cardiomyocyte apoptosis.

The effect of MA on cardiomyocyte apoptosis, an important component of cardiac remodeling leading to heart failure, was analyzed (19–22). Our data using TUNEL staining demonstrated that MA induces apoptosis in NRVMs. With varying concentrations (0, 0.5, 1.0 or 1.5 mM) of MA treatment, the apoptotic rate increased as the MA concentration increased. A significant increase in the apoptotic rate was detected after the NRVMs were treated with 1.5 mM MA (Fig. 1).

Identifying proteins associated with MA-induced apoptosis and silencing them is an essential step in developing therapeutics. In a previous study, our laboratory identified proteins associated with the pathogenesis of MA-induced toxicity by proteomic profiling in the rat brain, including oxidative stress, degeneration, apoptosis, mitochondrial pathway and energy metabolism (31). To probe more possible proteins, our laboratory also performed gene expression microarray analysis and revealed that IGFBP5 was significantly upregulated following MA exposure (data not shown).

Regulation of IGFs by IGFBP5 was known to affect cell proliferation, differentiation and survival. In particular, IGFBP5 has cell-type and tissue-type specific effects and its role in cell growth is complicated. IGFBP5 affects cell survival and apoptosis in a variety of cells, and the response to IGFBP5 depends on the cell type. IGFBP5 induces apoptosis in mammary epithelial cells (24), breast cancer cells (32) and osteosarcoma cells (33) but prevents apoptosis in neuroblastoma cells (34), C2 myoblasts (35) and human stellate cells (36). The increased expression of IGFBP5 may be involved in MA-induced toxicity, therefore we further postulate that IGFBP5 is one of the novel target involved in MA-induced apoptosis.

The results from the present study provided evidence that IGFBP5 expression at the transcriptional and translational levels were increased by MA treatment (Fig. 2), which is consistent with our hypothesis that IGFBP5 is involved in MA-induced cardiotoxicity. The mRNA and protein levels of IGFBP5 increased in a dose-dependent manner in NRVMs, and were significantly upregulated following treatment with 1.5 mM MA. The concentration of 1.5 mM MA was selected for our subsequent studies. To the best of our knowledge, this is the first study to report the increased expression of IGFBP5 in MA-treated NRVMs.

Previous studies have demonstrated that the expression of IGFBP5 is implicated in cardiovascular disease and even heart failure, which further suggests a novel role for IGFBP5 in the heart (37–40). Silencing of IGFBP5 expression allowed examination of its distinct cellular, physiological and pathophysiological functions (38,40). Synthetic siRNA targeting of the IGFBP5 gene was applied to silence IGFBP5 expression. Our data demonstrated that synthetic siIGFBP5 could efficiently knockdown IGFBP5 expression, and the nonspecific silencing control siNC demonstrated no effect on the expression of IGFBP5 (Fig. 3). Observations from the present study demonstrated that MA significantly increased the expression of IGFBP5 compared with the group not treated with MA. The efficient knockdown of IGFBP5 expression also presented in NRVMs with MA treatment was preceded by siIGFBP5 transfection (Fig. 3B).

IGFBP5 may induce or inhibit apoptosis in a variety of cells, and in the present study, the link between IGFBP5 and apoptosis induced by MA in NRVMs was observed. IGFBP5 was found to trigger apoptosis of NRVMs following exposure to MA. Furthermore, the results from the TUNEL assay demonstrated that silencing IGFBP5 expression protected NRVMs from MA-induced apoptosis to a certain degree (Fig. 4). IGFBP5 knockdown attenuated MA-induced apoptosis of cardiomyocytes. Our findings are consistent with the hypothesis that IGFBP5 may be involved in the loss of cardiomyocytes via apoptotic processes, and this evidence further suggested that IGFBP5 may have an important pathological role in MA-induced cardiotoxicity. However, the mechanisms contributing to this protective effect require further elucidation in future studies.

Activation of the caspase family is known to be a crucial mechanism for inducing apoptotic death signals. Pro-apoptotic signals trigger a cascade of caspases, which lead to the cleavage of a set of proteins, resulting in disassembly of the cell (41,42). Caspase-3 is the primary activator of apoptotic DNA fragmentation (43). The present study indicated that MA increased the expression of caspase-3. Silencing IGFBP5 with siRNA significantly suppressed the expression of caspase-3 in NRVMs following MA treatment. This indicated that the suppression or induction of apoptosis was mediated by a caspase-3-dependent pathway.

In conclusion, it is clear that IGFBP5 is involved in MA-induced apoptosis. The present study investigated cardiomyocyte apoptosis and the expression of IGFBP5 in MA-exposed NRVMs. To the best of our knowledge, the present study provided the first evidence that IGFBP5 is a potential regulator of MA-induced apoptosis. Additionally, silencing the expression of IGFBP5 is able to significantly protect cardiomyocytes from MA-induced apoptosis. Accordingly, the present study suggested that IGFBP5 is a potential target for the treatment of MA-induced apoptosis. It was further postulated that IGFBP5 provides a new mode for the regulation of apoptosis associated with MA-associated cardiac diseases.
